# A Method for Repeated, Longitudinal Sampling of Individual *Aedes aegypti* for Transmission Potential of Arboviruses

**DOI:** 10.3390/insects12040292

**Published:** 2021-03-27

**Authors:** E. Handly Mayton, Heather M. Hernandez, Christopher J. Vitek, Rebecca C. Christofferson

**Affiliations:** 1 Department of Pathobiological Sciences, School of Veterinary Medicine, Louisiana State University, Baton Rouge, LA 70803, USA; emayto1@lsu.edu; 2 Center for Vector-Borne Diseases, The University of Texas Rio Grande Valley, Edinburg, TX 78539, USA; hhernandez@hivresearch.org (H.M.H.); christopher.vitek@utrgv.edu (C.J.V.); 3 Center for Computation and Technology, Louisiana State University, Baton Rouge, LA 70803, USA

**Keywords:** vector competence, extrinsic incubation period, EIP, *Aedes aegypti*, arbovirus, transmission

## Abstract

**Simple Summary:**

Mosquito-borne viruses, such as Zika virus (ZIKV), remain a major public health concern worldwide. Vector competence is defined by the ability of a vector (mosquito) to become infected by and subsequently transmit a virus. Not all species of mosquitoes will transmit the same viruses; therefore, it is imperative that we continue to study mosquito–virus pairings in order to assess risk of transmission in different areas. Traditionally, a competent vector is determined by a high proportion of infectious saliva at terminal time points. However, a multitude of factors, such as mosquito biting habits and time, will have an impact on vector competence. We herein present a novel method for measuring biting habits and ZIKV transmission over time. To do this, we offered individual mosquitoes a bloodmeal (180 μL) every other day from 9 to 24 days post-exposure. Biting behavior was recorded as either probing, blood fed, or no bite; the bloodmeal was then collected and tested for the presence of ZIKV. Our results were successful in measuring behavior and viral transmission over time, and demonstrated variation among individual mosquitoes for both biting behavior and the amount of virus expectorated over time. Our results highlight the need for continued investigation into the complexity of vector competence, and we offer a method to aid in such investigations.

**Abstract:**

Mosquito-borne viruses are the cause of significant morbidity and mortality worldwide, especially in low- and middle-income countries. Assessing risk for viral transmission often involves characterization of the vector competence of vector–virus pairings. The most common determination of vector competence uses discreet, terminal time points, which cannot be used to investigate variation in transmission aspects, such as biting behavior, over time. Here, we present a novel method to longitudinally measure individual biting behavior and Zika virus (ZIKV) transmission. Individual mosquitoes were exposed to ZIKV, and from 9 to 24 days post-exposure, individuals were each offered a 180 μL bloodmeal every other day. Biting behavior was observed and characterized as either active probing, feeding, or no bite. The bloodmeal was then collected, spun down, serum collected, and tested for ZIKV RNA via qRT-PCR to determine individuals’ vector competence over time. This included whether transmission to the bloodmeal was successful and the titer of expectorated virus. Additionally, serum was inoculated onto Vero cells in order to determine infectiousness of positive recovered sera. Results demonstrate heterogeneity in not only biting patterns but expectorated viral titers among individual mosquitoes over time. These findings demonstrate that the act of transmission is a complex process governed by mosquito behavior and mosquito–virus interaction, and herein we offer a method to investigate this phenomenon.

## 1. Introduction

Vector-borne viruses remain a major cause of morbidity in low- and middle-income countries and have been making incursions into more temperate regions recently [1,2,3]. Because there is no treatment available for many of these viruses, determining the factors that promote arboviral transmission, emergence, and expansion is critical for predicting and controlling the impact on human and animal health. Dengue virus (DENV), chikungunya virus (CHIKV), and Zika virus (ZIKV) are transmitted by *Aedes aegypti*, an urban-dwelling mosquito widespread throughout tropical and subtropical areas [4,5,6,7,8]. The viruses this vector species transmits are responsible for large outbreaks affecting millions of people every year [9,10,11,12,13]. Complete understanding of the transmission systems of these arboviruses provides insight into the spread of the virus, especially when parameterizing prediction models that may be used in decision-making [14,15].

Vector competence is the intrinsic susceptibility of a vector species to infection with and subsequent transmission of a pathogen [9,15,16,17]. In a study on which Dr. William Black is the senior author, the importance of vector competence is explained as follows: “Understanding the relative vector competence of mosquitoes at the species, population, and individual levels is critical to the study of vector biology and the success of future vector-borne disease control programs” [18]. Measures of vector competence have evolved since first being included in the vectorial capacity equation in the late 20th century [16,19]. Vector competence has been determined by calculating either the proportion of vectors that are infected, that have a disseminated infection in the legs, or have detectable viral particles in forcibly collected saliva [16,20]. Usually, these measures are done at discrete, systematic time points, which may or may not accurately capture the process of vector competence [21,22]. For example, recent studies have demonstrated that discrete, terminal sample strategies do not capture the impact of individual heterogeneity on transmission efficiency [23,24,25,26] and that there are a multitude of factors that govern the ultimate success of transmission of an arbovirus, including mosquito behavior and within-virus kinetics [8,15,27].

While traditional ways of measuring vector competence are essential in determining successful vector–virus systems, the impact of these other factors, along with the impact of individual mosquito heterogeneity, must be investigated in order to further describe the transmitting population [28]. Here, we present a method of measuring transmission potential that longitudinally samples the same individual mosquitoes, capturing biting behavior and transmission capability over time, as well as heterogeneity in viral output from single mosquitoes. We use a model system of Rockefeller colony mosquitoes, field-derived mosquitoes, and ZIKV to demonstrate the method and describe the output data.

## 2. Materials and Methods

### 2.1. Cell Culture and Virus

ZIKV strain PRVABC59 (Asian lineage), which was isolated from human serum in Puerto Rico in 2015, was provided by Dr. Barbara Johnson at the US Centers for Disease Control and Prevention. Prior to use, the viral stock was passaged four times in Vero cells. On the fourth passage, cells were inoculated onto Vero cells at a multiplicity of infection (MOI) of 1. Supernatant was collected at 4 days post inoculation (dpi) and titer was determined by a neutral red plaque assay and qRT-PCR as previously described [29]. Virus was passaged onto Vero cells before being exposed to mosquitoes, as frozen virus has been shown to negatively affect mosquito susceptibility [30,31]. Supernatant was collected at 4 dpi and titer was determined using qRT-PCR before being used the same day for exposure. Titers were matched across all experiments as ~5 × 10^7^ pfu/mL as previously described [23,29].

### 2.2. Viral Quantification and Testing

RNA extraction was performed using the 5× MagMax96 viral nucleic acid isolation kit (Applied Biosystems, Foster City, CA, USA) and the KingFisher Flex (Thermo Fisher). Viral RNA was detected and quantified by qRT-PCR, using the SuperScript III Platinum Taq kit (Invitrogen) and the Roche LightCycler 96 as previously described [23,32]. A standard curve was run on all plates, with the lowest detectable dilution being our limit of detection (LoD). Any samples between our LoD Cq value and a Cq of 40 were inoculated onto Vero cells for confirmation of replicating virus. A neutral red plaque assay was used to titer our viral stock and indicated samples.

### 2.3. Mosquito Exposure and Maintenance

Lab-strain *Ae. aegypti* (Rockefeller) and fifth generation (F5) field-derived *Ae. aegypti* collected from southern Texas were used in this experiment. The Rockefeller strain was provided by Dr. Daniel Swale of the Louisiana State University Entomology Department, Baton Rouge, LA [23]. For field-derived *Ae. aegypti*, eggs were collected using oviposition traps in 7 cities across the Texas/Mexico border. Field collected eggs (F0) were hatched in a 1 g/L aerated nutrient broth mixture and reared to adult emergence in larval rearing pans stored at 23.9 degrees Celsius with 1:1 liver powder as needed. Once pupated, adults were moved to an environmental chamber kept at 24.6 °C and 70% relative humidity and a 16:8 light/dark cycle. One week after emergence, female mosquitoes were fed defibrinated cow blood using a Hemotek artificial feeding system (Hemotek, Blackburn, UK) and allowed to oviposit on oviposition papers in cages. These F1 generation eggs were provided to our lab and were reared in the same manner in the laboratory for four more generations. Generation five (F5) were used for experimental purposes here.

At 3–5 days post-emergence, mosquitoes were starved of sugar solution for 24 h before being exposed for 45 min to a ZIKV bloodmeal containing 2 mL whole bovine blood in Alsevers (Hemostat Labs, Dixon, CA, USA) and 1 mL viral supernatant using the Hemotek artificial feeding system with a 3 mL reservoir. Mosquitoes were then cold anesthetized and engorged females were sorted into new cartons. Cotton soaked with 10% sugar solution was provided for all mosquitoes *ad libitum.* Mosquitoes were housed at 28 °C, 16:8 light/dark schedule, and 80% relative humidity [33]. Wet oviposition paper was provided in each canister and carton and was rehydrated once per day.

### 2.4. Traditional Vector Competence Assay

ZIKV-exposed mosquitoes were sampled (*n* = 15–20) at corresponding time points—10, 14, and 18 days post exposure (dpe) for Rockefeller with an additional time point of 24 dpe from the individual cohort. Similarly, traditional vector competence included 10, 15, and 24 dpe for field-derived mosquitoes. These mosquitoes were not offered additional bloodmeal between exposure and terminal sampling. Infectious rates were determined by the presence of ZIKV in the saliva. Mosquitoes were cold anesthetized and placed on a cold pan before removing the legs and wings. Saliva was then collected via forced salivation by placing the proboscis into a micropipette tube containing 35 μL of fetal bovine serum (FBS) with 3 mmol/L ATP for 30 min as previously described [34]. Tip contents were then ejected into 100 μL of BA-1 (1% bovine serum albumin in M199X) media. RNA was extracted and qRT-PCR was performed on all samples as described above. In order to confirm the presence of replicating virus in the saliva, 50 μL of sample was inoculated onto 6-well plates of confluent Vero cells. Plates were rocked for 30 min at room temperature before 1.5 mL of M199X + 10% FBS, 2% antibiotic-antimycotic was added. Plates were observed for the presence of cytopathic effect (CPE) and supernatant was collected at 3- and 7-dpi and tested for the presence of viral RNA via qRT-PCR to confirm positive growth and thus the presence of infectious virus.

### 2.5. Limit of Detection

We compared the limits of viral recovery and detection using a known amount of virus and Hemotek reservoirs without having been offered to a mosquito. First, 180 μL of blood was spiked with 10 μL of the ZIKV viral stock described above at varying titers (10^4^–10^−1^ pfu/100 μL) and placed into Hemotek reservoirs. The Hemotek reservoirs were placed on the feeding system, which heats the reservoirs to 37 °C, for 45 min in order to mimic the conditions during blood offering to mosquitoes. Serum was collected and viral concentration determined via qRT-PCR. To determine agreement between qRT-PCR results and infectious viral particles, the same serum collections were plaqued using a neutral red assay.

### 2.6. Longitudinal Sampling

**Figure 1:**
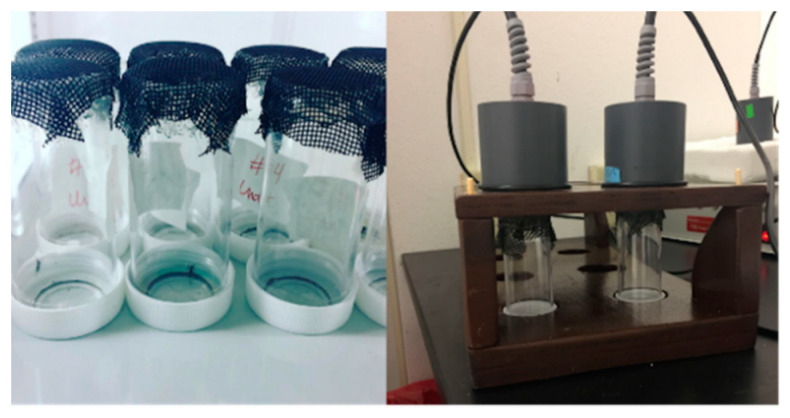
Experimental setup. Individual mosquitoes were housed in plastic canisters covered with fiberglass screen (**left**) and offered blood using the Hemotek artificial feeding system with the 300 μL reservoir and a custom stand (**right**).

Twenty-four hours before the start of the experiment, individual mosquitoes were placed into a clear, plastic canister (Figure 1). For our proof-of-principal trial with Rockefeller colony mosquitoes, one group of twelve mosquitoes was used every other day. We expanded this with the field-derived mosquitoes by assaying two staggered cohorts of individuals, one group observed/tested on even days, the other group on odd days. The tops of the canisters were removed and replaced with black fiberglass screen to avoid tearing the parafilm covering the Hemotek reservoir. Starting at 9 dpe (field-derived Group 1) or 10 dpe (Rockefeller/field-derived Group 2), mosquitoes were each provided an individual bloodmeal using a 0.3 mL reservoir containing 180 μL of bovine blood in Alsevers (see above). Blood was provided for 45 min at 37 °C. During the 45 min, behavior was observed by looking through the canister and/or lifting the reservoir and looking through the top of the canister to observe probing behavior at 1, 20, and 45 min as in [23]. Mosquitoes were classified as down (no probing or red abdomen observed), probed (exhibited probing behavior, no red abdomen observed), or fed (red abdomen observed).

After 45 min, blood was removed from the reservoirs by piercing the parafilm with a pipette tip, removing the blood with the pipette, and placing into individual microcentrifuge tubes. Tubes were centrifuged for 6 min at 4000 rpm at 4 °C to separate the serum. Serum was removed and placed in a new tube for further testing. This was done until 24 dpe, determined by previously observed vector competence studies and average time to death [23,33,34]. Collected serum was tested for the presence of viral RNA via qRT-PCR. In order to confirm the presence of replicating virus in the serum, 25 μL of sample was inoculated onto 12-well plates of confluent Vero cells. Plates were rocked for 30 min at room temperature before 1.5 mL of M199X + 10% FBS, 2% antibiotic-antimycotic was added. Plates were observed for the presence of CPE and supernatant was collected at 3- and 7-dpi and tested for the presence of viral RNA via qRT-PCR to confirm positive growth and thus the presence of infectious virus. On the final day of the experiment, mosquitoes that survived were force salivated and processed as described for traditional vector competence.

We wanted to determine whether this method could be used to test whether the proportion of probing events vs. feeding events resulting in transmission was significantly different. To do this, we used a chi-square test of proportion (function prop.test, R version 3.5.3), with a confidence level of 95%. As a means of determining general reproducibility (lack of variation between replicates), we compared the proportions of overall biting events and transmission events within the two field-derived groups using the chi-square test of proportion as above.

## 3. Results

### 3.1. Model System Development

#### 3.1.1. In Vitro Limit of Detection

To determine the limit of detection of our method, we first measured the recoverability of virus from sera using a controlled scenario. When we compare the viral concentration from recovered sera via qRT-PCR and paired plaque assay, there was complete agreement at dilutions 10^0−2^. At higher viral concentrations, quantification of the plaque assay was hampered by too many plaques to count. We did determine that our limit of detection was 1 pfu/100 μL. However, our qRT-PCR assay was more sensitive and detected down to 0.1 viral RNA copies/100 μL (Table S1). Based on these results, we determined our method to be sensitive and moved forward with the experiment using the qRT-PCR to determine viral concentration and delineate between pfu/volume versus RNA equivalents/volume.

#### 3.1.2. Vector Competence by Traditional Measures

Rockefeller mosquitoes were terminally tested for the presence of ZIKV in the saliva at 10, 14, 18, and 24 dpe. Forced saliva results revealed 0% of mosquitoes transmitted at 10 dpe, 26.7% transmitted at 14 dpe, 46.7% transmitted at 18 dpe, and 87.5% at 24 dpe. All positive qRT-PCR samples were confirmed infectious by observation of viral growth in vitro. Titers of forced saliva and in vitro collections from 24 dpe are reported here (Table S2). This indicates moderate to high vector competence as per traditional vector competence, which is consistent with previous studies [23,30].

#### 3.1.3. Individual, Longitudinal Vector Competence Method

**Figure 2:**
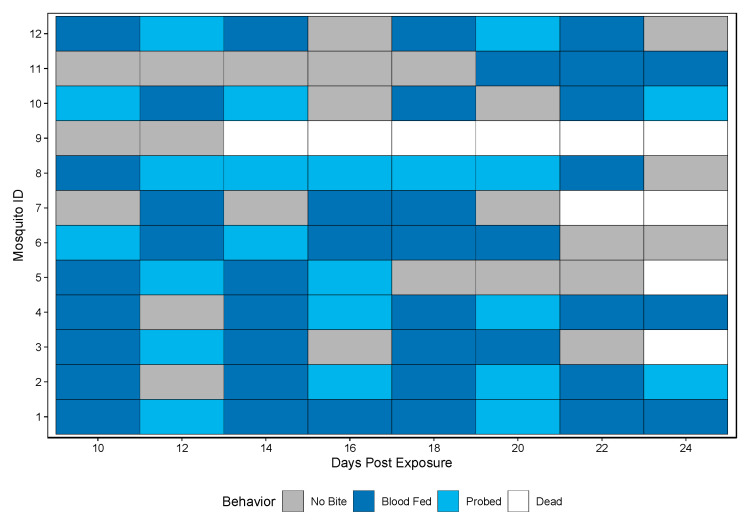
Biting behavior of Rockefeller individuals over the study period. Each row represents one mosquito across days post exposure. Each shaded square represents a potential transmission event, with mosquitoes being classified as no biting (grey), probing (light blue), and blood fed (dark blue). White squares indicate no opportunity (dead).

A novel method was developed to assess vector competence, extrinsic incubation period (EIP), and biting habits at the individual mosquito level. Biting behavior was observed when bloodmeals were offered every other day starting at 10 dpe and ending at 24 dpe. Biting behaviors were recorded as either blood fed or probed. Over the course of the study period, 11/12 Rockefeller mosquitoes bit (either blood fed or probed) and all 11 bit more than once (either blood fed or probed) (Figure 2).

**Figure 3:**
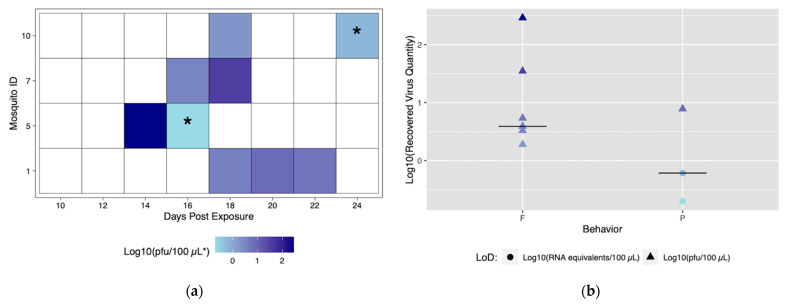
Viral titers expectorated with each successful transmission event by Rockefeller individuals. Only mosquitoes which had successful transmission over time are shown. A successful transmission event is defined by a positive serum sample via qRT-PCR and growth on Vero cells. (**a**) Each shaded square represents a transmission event, with white squares representing no detectable virus. Other squares are scored from lowest viral titer (light blue) to highest viral titer (navy). (**b**) Viral titers present in serum were compared for blood fed (F) vs. probed (P). Viral titers range from lowest detectable titer (light blue) to highest detectable titer (navy). Asterisks (*) indicate where recovered quantity is below the limit of detection from our sensitivity analysis and reflect qRT-PCR values of genome equivalents (viral RNA copies/100 μL) rather than pfu/100 μL.

Despite robust biting habits, only four individuals from the Rockefeller colony successfully transmitted (ID# 1, 5, 7, and 10), and of those all four transmitted more than once with a total number of nine transmission events (Figure 3a). From this method, we are able to discern time to first transmission, which was 14 dpe (Mosquito #5). In addition, it was possible to observe and characterize repeated transmission from the same mosquito specimen. Mosquito #1 had the most transmission events, with three starting at 18 dpe (Figure 3a).

The role of different biting behaviors and the subsequent transmission was observed. There was a total of three transmission events with probing and seven associated with blood feeding from Rockefeller mosquitoes (Figure 3a). We calculated the proportion of probing and feeding events that resulted in transmission as 13.6% and 15%, respectively. There was not a significant difference between transmission proportion relative to type of behavior (*p* > 0.05). The range of recovered viral quantities from serum collections was 0.2 viral RNA copies/100 μL (below in vitro limit of detection), and 1.9 pfu/100 μL to 290 pfu/100 μL (within in vitro limit of detection). Interestingly, we observed variability in output from the same mosquito over different transmission events (Figure 3b) (Table S3). Both the lowest (probing) and highest (blood feeding) recovered virus quantity was from the same mosquito (ID# 5) (Figure 3a).

**Figure 4:**
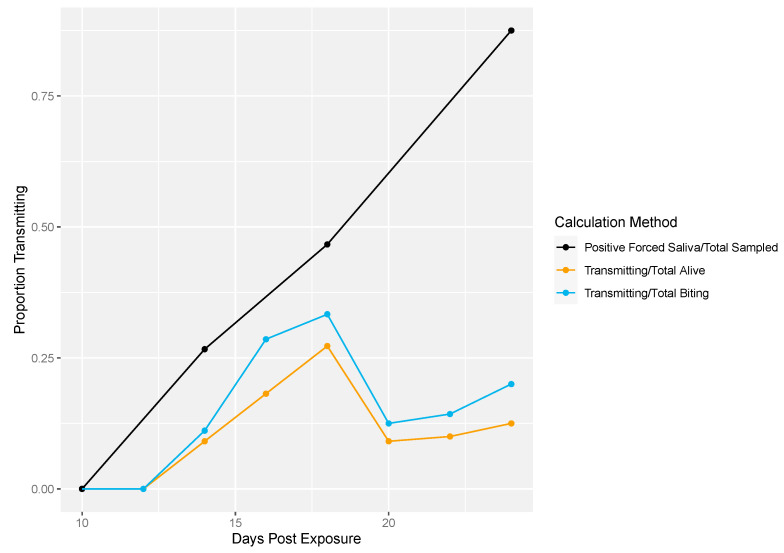
Rockefeller transmission profile. Traditional vector competence was calculated as the proportion of mosquitoes with positive forced saliva samples divided by the number sampled (black line). From the longitudinal sampling method, the total number of transmitting mosquitoes over the total number of living mosquitoes was calculated per sampling day (yellow line). Lastly, the total number of transmitting mosquitoes over the total number of mosquitoes that bit was calculated per sampling day (blue line).

Next, we demonstrated the differences in traditional vector competence measures to findings from our longitudinal sampling methodology (Figure 4). There was no transmission at 10 dpe in either method (Figure 4). The proportion of transmission events observed from the longitudinal sampling method was calculated two different ways: (1) as the proportion of mosquitoes that successfully transmitted over the total number of living mosquitoes per sampling day and (2) the number of mosquitoes that successfully transmitted over the total number of mosquitoes that bit per sampling day. Overall, lower proportions of transmission events (calculated either way) were observed compared to the proportion infectious mosquitoes measured by traditional vector competence (Figure 4). In the longitudinal sampling method, we found that the proportion of mosquitoes that transmitted out of biting mosquitoes was higher than the proportion that transmitted out of total mosquitoes, indicating that the denominator (and thus transmitting proportion) is sensitive to inclusion of biting behavior.

### 3.2. Application of Method to Field-Derived Mosquitoes

**Figure 5:**
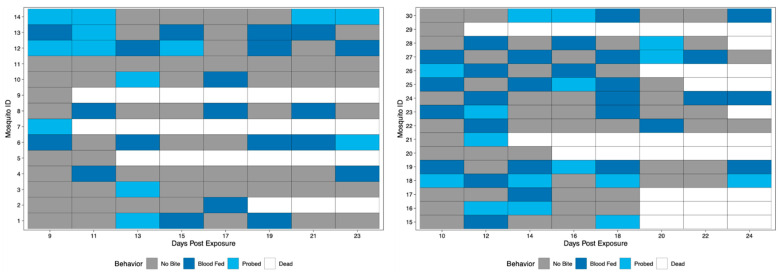
Biting behavior of field-derived individuals over the study period. Each row represents one mosquito across days post exposure. Each shaded square represents a potential transmission event, with mosquitoes being classified as no biting (grey), probing (light blue), and blood fed (dark blue). White squares indicate no opportunity (dead). Mosquito IDs 1–14 represent Group 1 (left), while IDs 15–30 represent Group 2 (right).

Field-derived mosquitoes were terminally tested for the presence of ZIKV in the saliva at 5, 10, 15, and 24 dpe. Results revealed 0% of mosquitoes had viral detection at 5 and 10 dpe, 5% had detection at 15 dpe, and 62.5% had detection at 24 dpe. Again, all positive qRT-PCR samples were tested for infectious virus by observation of viral growth in vitro (Table S2). Biting behavior was observed when bloodmeals were offered every other day starting at 9 dpe (field-derived Group 1) or 10 dpe (field-derived Group 2) and ending at 24 dpe to get complete coverage of all days of the study of field-derived mosquitoes. When compared, biting frequencies between Group 1 and Group 2 of the field-derived mosquitoes were not significantly different; therefore, field-derived groups were combined and will be described as one population of 30. Over the course of the study period, 25/30 mosquitoes bit, and 20/25 bit more than once (Figure 5). When compared via chi-square test of proportions, Rockefeller mosquitoes exhibited a significantly higher proportion of biting behavior (72.1%) than field-derived mosquitoes (42.1%) (*p*-value < 0.05).

**Figure 6:**
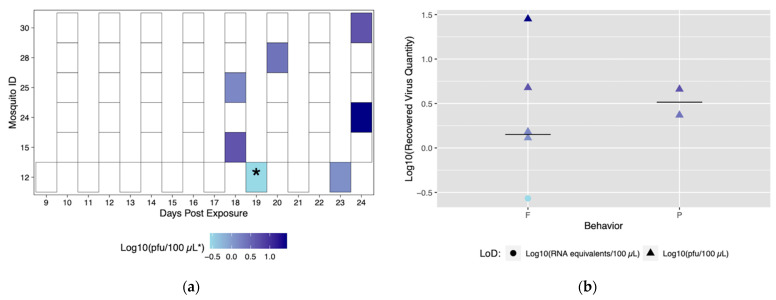
Viral titers expectorated with each successful transmission event by field-derived individuals. Only mosquitoes which had successful transmission over time are shown. A successful transmission event is defined by both a positive serum sample via qRT-PCR and growth on Vero cells. (**a**) Each shaded square represents a transmission event, with white squares representing no detectable virus. Other squares are scored from lowest viral titer (light blue) to highest viral titer (navy). (**b**) Viral titers present in serum were compared for blood fed (F) vs. probed (P). Viral titers range from lowest detectable titer (light blue) to highest detectable titer (navy). Asterisks (*) indicate where recovered quantity is below the limit of detection from our sensitivity analysis and reflect qRT-PCR values of genome equivalents (viral RNA copies/100 μL) rather than pfu/100 μL.

Six field-derived individuals successfully transmitted (ID# 12, 15, 24, 25, 28, 30), but there were only seven transmission events (Figure 6a). Time to first transmission occurred at 18 dpe, and only one mosquito had more than one successful transmission event (Figure 6a). Of the seven successful transmission events, five resulted from feeding behavior and two events resulted from probing behavior. Similar to the Rockefeller colony, blood feeding behavior yielded a higher maximum titer compared to probing behavior (Figure 6b). When compared, the proportion of transmission events by feeding (71.4%) was significantly higher than events by probing (28.6%) (*p* < 0.05). Mosquito #12 was the only mosquito to successfully transmit more than once. Again, titers were variable among transmission events. Field-derived mosquitoes expectorated viral quantities ranging from 0.2 viral RNA copies (below in vitro limit of detection), and 1.5 to 28.3 pfu/100 μL (Table S3).

**Figure 7:**
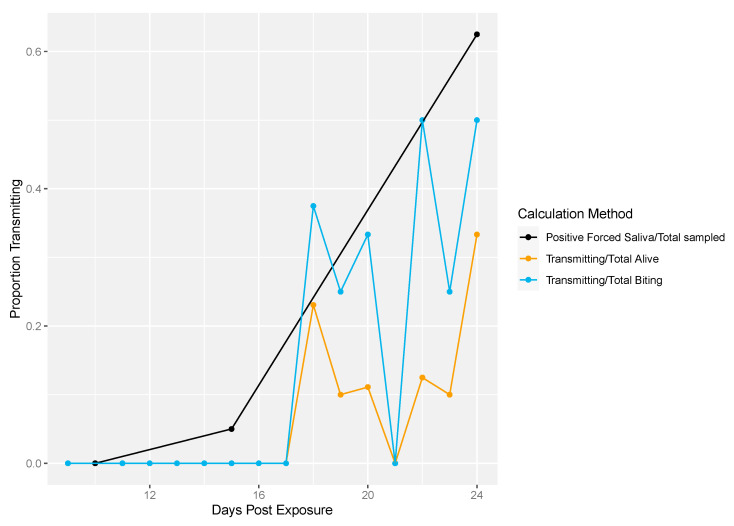
Field-derived transmission profile. Traditional vector competence was calculated as the proportion of mosquitoes with positive forced saliva divided by the number sampled (black line). The total number of transmitting mosquitoes over the total number of living mosquitoes was calculated per sampling day (yellow line). Lastly, the total number of transmitting mosquitoes over the total number of mosquitoes that exhibited biting behavior was calculated per sampling day (blue line).

Figure 7 shows the differences in traditional vector competence measures and the longitudinal sampling method for field-derived mosquitoes. Again, the traditional measure reached higher transmission rates compared to the longitudinal sampling measures (Figure 7).

## 4. Discussion

*Ae. aegypti* are unique in that they take multiple bloodmeals during a gonotrophic cycle [35,36]. Being an urban mosquito, they are often present in or near households, making it likely to bite humans more than once [37,38]. Traditional vector competence tells us the subset of mosquitoes that are capable of transmitting arboviruses. However, actual transmission is a function of several other conditions. Here, we have developed a method which can account for the interaction of some of the vector traits that define these conditions and the subset of mosquitoes that do the transmitting; namely, biting behavior, vector competence, and EIP at one time. Due to the many factors affecting vector competence, such as geographic location, viral strain, and mosquito population/species, it is imperative that we continue exploring the heterogeneity of transmission potential both at the population and individual levels [2,17,30,31]. Further, modification of this method could target time points of the same mosquito to get more traditional measures (% at discreet time points) to determine the distribution of EIP in a population after exposure.

Importantly, this method successfully reveals the heterogeneity of transmission potential among individuals. The relationship between biting and viral output were observed. Overall, titers of recovered serum ranged from 10^2^–10^−1^, which is consistent with a previous study of ZIKV in *Ae. aegypti* [2]. Our data suggests that those that fed tended to have higher viral titers recovered from the serum than those that probed, which was previously observed in *Culex* spp. [39]. Congruency with these two studies suggests our method will be a useful tool for assessing vector competence and testing hypotheses regarding viral transmission at the individual-mosquito level. For example, we observed one mosquito having both the highest and the lowest viral titer output, associated with a bite and a probing event, respectively. Further, we observed that one mosquito had an “empty” feeding event between transmission events, while other transmitters had consistent transmission. Further, average viral titer of serum collections differed between the two colonies, suggesting differences of viral output at both the individual and population level. Differences in biting frequency between the Texas and Rockefeller mosquitoes were noted, with biting proportions significantly lower for field-derived Texas mosquitoes compared to the Rockefeller colony. This is not surprising, as lab colonies are likely adapted to lab conditions, which is why we chose to validate the method in field-derived mosquitoes [40]. This longitudinal method is thorough enough to detect these differences and thus allows for further hypothesis testing regarding the mechanisms behind this phenomenon and other heterogeneity observed. Other methods have also investigated vector competence in longitudinal ways, highlighting the importance of this research [41,42]. Of course, with an artificial system, there is a lack of biological cues associated with feeding [43,44,45]. Although our method uses an artificial system, this makes it both accessible and cost effective while longitudinally sampling individuals for virus transmission in the context of mosquito behaviors.

Traditional vector competence is a cumulative measure, which is monotonically increasing and often described by a logistic function [24,46]. In contrast, our results are highly variable, indicating the process of transmission is likely heterogeneous at the population and individual levels. For example, when we further consider cumulative transmission events as the proportion of transmission events over cumulative biting events, these were not significantly different between Rockefeller and field-derived (14.5% vs. 16.7%, respectively, *p* > 0.05), despite field-derived mosquitoes having a lower overall biting frequency. This suggests that continued study is needed to elucidate the functional relationship between population bite frequency and transmission intensity.

The discrepancy between traditional vector competence and the results from the longitudinal sampling method could be due to several factors. First, our mosquitoes were offered multiple bloodmeals, which has been shown to increase vector competence for some arboviruses [26]. However, previous work from our laboratory showed contrasting results [23]. Second, our method takes into account mosquito behavior, which traditional vector competence measures cannot. Third, forced salivation assays by definition, compels salivation and all but guarantees virus recovery. The traditional assay does not account for the myriad of micro-processes that occur during mosquito contact with human hosts, including variability in saliva deposition, behaviors, and the possibility of inherent heterogeneity among mosquitoes. We hypothesize that this individual, longitudinal method provides a means to test how these and other factors define the successful contacts that result in transmission. Importantly, we successfully observed these differences, and were able to isolate virus from these biting events.

Traditional vector competence remains a crucial part of identifying vectors with the potential to support viral spread, and is an important step towards investigating intricate interactions between the vector, virus, and environment occurring at all stages of vector competence [47]. The novel method proposed herein can also be performed at limited, discrete time points, similar to traditional vector competence sampling methods, which would be more directly comparable to vector competence data output. However, our method—unlike cohort sampling—allows for observation of individual heterogeneity of metrics such as the extrinsic incubation period, viral titer output, and associated biting behavior of the mosquito. Further, longitudinal measurement of individual feeding opportunities, for example, can be used to determine not only post-exposure dynamics (transmission), but interrogate the role of pre-exposure behaviors that affect the infection and dissemination dynamics within cohorts (e.g., number of bloodmeals). Ultimately, this method can be used to ask nuanced questions about effectors of vector competence and transmission, such as the role of length of time probing, vector–virus interactions, and the role of environmental factors [48,49,50].

**Figure 8:**
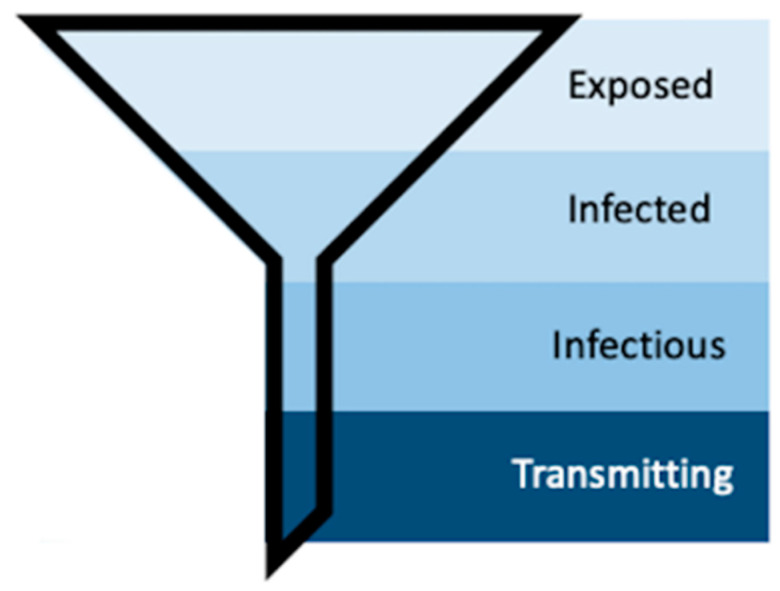
Schematic representing the process of vector competence. As susceptible mosquitoes progress through the stages of vector competence, the population funnels down into the transmitting population, the small group that our method aims to further describe.

Mosquito populations in arbovirus-endemic areas can be subset according to exposure (Figure 8). First, only a subset of the total mosquito population will become exposed to infectious individuals (“Exposed”). Second, a subset of these mosquitoes will become infected, meaning the infection will remain sequestered in the midgut (“Infected”) [16,51]. Third, some of these infected mosquitoes will develop disseminated infections—which is what is measured by traditional vector competence assays that use periphery tissues such as legs, wings, or heads to detect the presence of virus [15,30,31]. Forced saliva, while thought to be a better indication of truly infectious individuals, still only identifies that subset of mosquitoes that are infectious because the data inherently describes those mosquitoes that can transmit. Longitudinal sampling in the manner described herein includes the characteristics of individual mosquito behavior, which further funnels the population of mosquitoes into those that *do* transmit (Figure 8). Further, our method identifies repeat transmitters, which is impossible with terminal assays, and with this we can begin to investigate the concept of super-spreaders and the multitude of individual and heterogeneous vector–virus interactions that drive transmission.

## Data Availability

Not applicable.

## Supplementary Materials

The following are available online at https://www.mdpi.com/article/10.3390/insects12040292/s1, Table S1: Limit of detection for novel assay for qRT-PCR and neutral red plaque assay. Bloodmeals were spiked with a known titer of virus and loaded into a Hemotek reservoir before incubating on an artificial feeding system for 45 minutes. Blood was collected from the reservoir and serum was titered using qRT-PCR. Additionally, serum was plaqued using neutral red and Vero cells. Five replicates were performed. Plaque counts are listed, with excessive plaques being classified as TTC (too many to count). Titers obtained via qRT-PCR are listed below, Table S2: Titers of individual forced saliva at 24 days post-exposure. Surviving mosquitoes at the end of the model system ex-periment were force salivated. Positive forced saliva samples were determined via qRT-PCR. Posi-tive individuals and their respective titers are listed below. To confirm the presence of replicating virus, 50 μL of remaining saliva was inoculated onto 6 well plates of confluent Vero cells. At 3 and 7 days post-inoculation, supernatant was collected and tested for viral replication via qRT-PCR for the positive growth, Table S3: Titers of collected serum and collections from Vero in-oculation. Serum collected from successful transmission events were titered via qRT-PCR. Colony, individual mosquito ID, and days post mosquito exposure at time of transmission event are listed below. For confirmation of replicating virus, 25 μL of remaining serum was inoculated onto 12 well plates of confluent Vero cells. At 3 and 7 days post-inoculation, supernatant was collected and tested for viral replication via qRT-PCR for the positive growth.
