# Supplementary material for: A Method for Repeated, Longitudinal Sampling of Individual Aedes aegypti for Transmission Potential of Arboviruses
Source: Insects. 2021 Mar 27;12(4):292. doi: 10.3390/insects12040292 (PMC8065608; doi:10.3390/insects12040292)
Supplement: Supplementary file 1 [file insects-12-00292-s001.pdf]

Supplemental Table 1. Limit of detection for novel assay for qRT-PCR and neutral red plaque assay. Bloodmeals were spiked with a known titer of virus and loaded into a Hemotek reservoir before incubating on an artificial feeding system for 45 minutes. Blood was collected from the reservoir and serum was titered using qRT-PCR. Additionally, serum was plaqued using neutral red and Vero cells. Five replicates were performed. Plaque counts are listed, with excessive plaques being classified as TTC (too many to count). Titers obtained via qRT-PCR are listed below.

Assay						
Plaque Assay	Dilution (pfu/100 μL)	Rep 1	Rep 2	Rep 3	Rep 4	Rep 5
	10^4	TTC	TTC	TTC	TTC	TTC
	10^3	TTC	TTC	TTC	TTC	TTC
	10^2	59	15	14	17	31
	10^1	2	4	4	7	8
	10^0	0	0	0	1	3
	10^{-1}	0	0	0	0	0
qRT-PCR	Dilution (pfu/100 μL)	Rep 1	Rep 2	Rep 3	Rep 4	Rep 5
	10^4	4.2×10^4	9.9×10^4	1.2×10^4	1.7×10^4	2.1×10^4
	10^3	5.8×10^3	4.5×10^3	2.4×10^3	1.0×10^3	3.2×10^3
	10^2	7.7×10^2	1.2×10^3	1.2×10^3	9.1×10^2	7.1×10^2
	10^1	8.6×10^1	1.4×10^2	2.8×10^1	5.7×10^1	3.1×10^1
	10^0	1.1×10^1	4.4×10^0	3.9×10^0	1.0×10^1	9.5×10^0
	10^{-1}	8.6×10^{-1}	1.1×10^0	0	1.9×10^0	3.8×10^{-1}

Supplemental Table 2. Titers of individual forced saliva at 24 days post-exposure. Surviving mosquitoes at the end of the model system experiment were force salivated. Positive forced saliva samples were determined via qRT-PCR. Positive individuals and their respective titers are listed below. To confirm the presence of replicating virus, 50 μL of remaining saliva was inoculated onto 6 well plates of confluent Vero cells. At 3 and 7 days post-inoculation, supernatant was collected and tested for viral replication via qRT-PCR for the positive growth.

Colony	Mosquito ID	Titer of saliva (Viral RNA Copies/100 μL)	Replication in cell culture (Yes/No)
Rockefeller	1	7.7	Y
	2	0.9	Y
	6	45	Y
	8	6.8	Y
	10	0.4	Y
	11	240	Y
	12	3.0	Y
Field-derived	1	1.7	Y
	4	1.1	Y
	6	5.1	Y
	10	7.2	Y
	11	3.5	Y
	12	70	Y
	13	0.8	Y
	19	9.4	Y
	24	13	Y
	30	1.8	Y

Supplemental Table 3. Titers of collected serum and collections from Vero inoculation. Serum collected from successful transmission events were titered via qRT-PCR. Colony, individual mosquito ID, and days post mosquito exposure at time of transmission event are listed below. For confirmation of replicating virus, 25 μL of remaining serum was inoculated onto 12 well plates of confluent Vero cells. At 3 and 7 days post-inoculation, supernatant was collected and tested for viral replication via qRT-PCR for the positive growth.

Colony	Mosquito ID	Days post-exposure	Titer of serum (Viral RNA Copies/100 μL)	Replication in cell culture (Yes/No)
Rockefeller	5	14	290	Y
	5	16	0.2	Y
	7	16	3.3	Y
	1	18	3.9	Y
	7	18	35	Y
	10	18	1.9	Y
	1	20	7.8	Y
	1	22	5.4	Y
	1	24	1.6	N
	10	24	0.6	Y
Field-derived	15	18	4.6	Y
	23	18	0.2	N
	25	18	1.5	Y
	12	19	0.7	Y
	28	20	2.3	Y
	8	21	0.3	N
	24	22	4.9	N
	12	23	3.1	Y
	24	24	28	Y
	30	24	4.8	Y
